# Physical Activity and Pain Perception in Residents Under Conditions of Chronic Hypoxia

**DOI:** 10.3390/oxygen5030011

**Published:** 2025-06-30

**Authors:** Margot Evelin Bernedo-Itusaca, Kely Melina Vilca-Coaquira, Ángel Gabriel Calisaya-Huacasi, Madeleyne Rosmery Cosi-Cupi, Stanley Rivaldo Leqque-Santi, Shantal Cutipa-Tinta, Alberto Salazar-Granara, Yony Martin-Pino Vanegas, Alcides Flores-Paredes, Shihui Guo, William Li, Moua Yang, Ginés Viscor, Ivan Hancco Zirena

**Affiliations:** 1 Facultad de Medicina Humana, Universidad Nacional del Altiplano, Puno 21000, Peru; 2 ACEM (Asociación Científica de Estudiantes de Medicina), UNA, Puno 21000, Peru; 3 Centro de Investigación en Medicina de Altura (CIMA), Facultad de Medicina Humana, Universidad de San Martín de Porres, Lima 15001, Peru; 4 Facultad de Educación, Escuela Profesional de Educación Física, Universidad Nacional del Altiplano, Puno 21001, Peru; 5 Division of Thrombosis and Hemostasis, Beth Israel Deaconess Medical Center and Harvard Medical School, Boston, MA 02108, USA; 6 Department of Anesthesia, Beth Israel Deaconess Medical Center, Boston, MA 02108, USA; 7 Harvard Medical School, Harvard University, Boston, MA 02115, USA; 8 Bloodworks Northwest Research Institute, Seattle, WA 98102, USA; 9 Division of Hematology and Oncology, Department of Medicine, University of Washington School of Medicine, Seattle, WA 98195, USA; 10 Sección de Fisiologia, Departament de Biologia Cel·lular, Fisiologia i Immunologia, Facultat de Biologia, Universitat de Barcelona, E-08028 Barcelona, Spain

**Keywords:** physical activity, pain tolerance, hypoxia, pain threshold, tourniquet test

## Abstract

**Background:**

Previous studies indicate that individuals who engage in regular physical activity have a higher pain threshold than those who do not exercise. However, it remains unclear how this phenomenon behaves in individuals exposed to chronic hypoxia. This study evaluates pain perception at high altitude between high-altitude natives who exercised regularly and those who did not practice physical activity.

**Methods:**

Eighty-four healthy volunteers aged 20 to 30 years old with a body mass index (BMI) within the normal range (18.5–24.9) residing in the city of Puno (3825 m) were recruited. The unilateral ischemia pain provocation test was used, applying pressure with a manual sphygmomanometer to generate transient ischemia in the arm while the patient opens and closes their hand. Onset, peak, and resolution times of pain, heart rate, and oxygen saturation were recorded.

**Results:**

The average time to pain onset in the right arm was 30.2 s ± 14.1 during light physical activity, whereas, during moderate physical activity, it increased to 32.5 s ± 15.4. In the left arm, the average time until pain sensation was 27.9 s ± 16.8 during light physical activity and increased to 34.6 s ± 18.5 with moderate physical activity. Regarding the progression of pain intensity, the average time to reach unbearable pain in the right arm was 54.1 s ± 16.4 during light physical activity and 53.8 s ± 19.6 during moderate physical activity; in the left arm, it was 53.0 s ± 19.6 during light physical activity, increasing to 59.3 s ± 24.5 during moderate physical activity.

**Conclusions:**

A more stable and slightly higher pain tolerance in the dominant arm was observed.

## 1. Introduction

The International Association for the Study of Pain (IASP) defines pain as “an unpleasant sensory and emotional experience associated with, or similar to that associated with, actual or potential tissue damage”, in addition to highlighting the characteristic of subjectivity and inclusion of cognitive and emotional aspects [1]. Pain is probably the most common symptomatic reason to seek medical consultation. All of us have suffered headaches, burns, cuts, and other pain causes at some time during childhood and adult life [2].

Regular and adequate physical activity (PA) brings multiple health benefits, including cardiovascular function improvement, body weight control, prevention of chronic diseases, and general well-being. PA also contributes to the prevention, among others, of cardiovascular diseases and cancer, in addition to improving people’s quality of life. Currently, PA is a non-pharmacological intervention commonly recommended to prevent and treat a variety of diseases, such as high blood pressure, and metabolic disorders, such as type II diabetes. Benefits of PA have been observed in diseases characterized by the presence of chronic pain, due to its hypoalgesic effect that is related to the type of PA and its duration [2-5], as evidenced by recordings of pain sensitivity before and after exercise. Isometric muscle contraction has been linked to the modulation of pain sensitivity in healthy subjects [6,7]. In healthy subjects, exercise-induced hypoalgesia (EIH) is often demonstrated after isometric exercises as an increase in pressure pain thresholds [8,9]. On the other hand, the analgesic effects of EMS-induced muscle contractions are primarily localized to the stimulated muscle tissues, rather than mediated by central pain modulatory mechanisms, as evidenced by quantitative sensory tests [10]. The potential effect of exercise on pain tolerance could be relevant for patients afflicted with chronic pain as a pain-coping strategy [11]. An additional consequence would be the increase in pain tolerance. Studies show the isolated beneficial outcome of physical activity on pain tolerance; however, there has been no conclusive evidence regarding the exact mechanisms of the underlying benefits. In addition, no studies have been conducted to establish the intensity and pattern of physical activity required to improve pain tolerance.

Another factor associated with a greater pain tolerance is chronic hypoxia [12], which occurs as a result of prolonged exposure to environmental low oxygen partial pressure. In addition to the altitude adaptation process, hypobaric hypoxia affects the ability to perform intense physical activity and alters the pain perception. Some studies suggested that chronic hypoxia can increase pain sensitivity, while others indicated that regular physical activity can improve pain tolerance [13,14]. Still, it is unclear whether the effects of exercise practice in subjects living under conditions of chronic hypoxia impact the perception of pain. In addition, it is not understood how physical activity can modify pain tolerance under conditions of prolonged exposure to hypobaric hypoxia.

In populations subjected to chronic hypoxia, there is a need to further investigate the relationship between physical activity and pain tolerance, which could have important implications for the promotion of healthy lifestyles and pain management in highland populations. In the present study, we have evaluated the difference in pain perception using an easy-to-execute method in a group of apparently healthy volunteers who permanently reside at high altitude (hypoxic environment). We have compared a group of people with a sedentary lifestyle versus another group with a regular level of moderate physical activity, intending to evaluate their potential differences in pain tolerance.

## 2. Materials and Methods

### Participants:

Eighty-four healthy volunteers were recruited and assigned to one of two groups—individuals who engaged in regular exercise (n = 47) and those who did not participate in physical activity (n = 37). The inclusion criteria for the study were healthy volunteers between 20 and 30 years old with a body mass index (BMI) within the normal range (18.5–24.9). For the exercise group, those who regularly carried out moderate physical activity were included, while the non-exercise group consisted of volunteers who did not regularly exercise. Exclusion criteria included, but were not limited to, musculoskeletal disorders, and any significant medical condition, such as neurological (schizophrenia, Alzheimer’s), cardiovascular, respiratory, or metabolic diseases (diabetes mellitus II, obesity). All participants had to be available for all of the study phases and gave their written informed consent before carrying out the test.

### Procedure:

Subjects were assessed in Puno (3827 m), their hometown. The following data were recorded: age, sex, weight, height, and body mass index. Systolic blood pressure (SBP), diastolic blood pressure (DBP), and heart rate (HR) were registered using a Ri-Champion digital blood pressure monitor (Rudolf Riester GmbH, Jungingen, Germany) with a measuring range of 30 to 280 mm Hg for blood pressure and a range of 40 to 200 beats per minute for heart rate. SatO_2_ was measured with a NELLCOR^®^ OXIMAX^®^ N-65 pulse oximeter (Digicare Biomedical Technology Inc., Boynton Beach, FL, USA), with a saturation resolution of 1% and a heart rate range of 30 to 235 beats per minute. Capillary blood samples were obtained from the pad of the finger of each participant. A puncture was performed with a sterile lancet to absorb a drop of blood in a microcuvette. The hemoglobin was measured with a Hb-201 hemoglobinometer (Hemocue, Ängelholm, Sweden), using the azidimethemoglobin method within a measurement range of 0 to 25.6 g/dL. Hematocrit (Hct) was measured in a HemataStat II microcentrifuge (EKF Diagnostics, Penarth, UK) on blood samples obtained by a fingertip puncture.

### Measurement of pain tolerance (Tourniquet ischemic test):

We used the tourniquet test, which is a simple and low-cost pain tolerance assessment technique that any trained healthcare personnel can appropriately apply. The tourniquet test is based on the neurophysiological response to pain induced by ischemia and nerve compression. During its application, the interruption of blood flow generates local ischemia, and the pressure exerted on the nerves activates nociresponsive neurons in the dorsal horn of the spinal cord. These neurons increase their spontaneous activity and expand their receptive fields, especially in areas close to the site of the tourniquet. This phenomenon reflects peripheral and central sensitization mechanisms, which intensify the perception of pain and make it more intense and persistent. Although its accuracy is limited, this technique represents a useful tool for the initial clinical evaluation of pain tolerance. Ischemic pain is elicited by the ischemic handgrip exercise (20 times) of the subject after the tourniquet is inflated around the upper arm. The test performance is measured in terms of elapsed time between the cessation of squeezing and the report of slight (threshold) and unbearable (tolerance) pain. The subject is in a sitting position with the arm resting on a table. The arm remains at the same level as the heart to place the manual blood pressure monitor with the lower edge 2 cm above the bend of the elbow. The cuff is insufflated up to 200 mmHg. Then, the patient opens and closes their hand rhythmically. A specific cadence for hand open–close was not established with a metronome, although it was similar between subjects, at around 1 Hz. With a stopwatch, the evaluator records the times of pain onset, when the pain becomes unbearable, and when the pain disappears. These timing data also allow us to calculate the exacerbation time (exacerbation = unbearable − onset) and the resolution time (resolution = disappearance − unbearable).

### Statistical analysis:

Collected data were analyzed using SPSS 30.0 software (package version), descriptive analysis, normality tests, comparisons between groups, and correlations to explore associations between variables were performed. Statistical significance was considered for *p* < 0.05. Data for all parameters were expressed as the arithmetic mean and standard deviation. The normality distribution of the datasets was determined with the Shapiro–Wilk test, which resulted in all variables showing a normal distribution. Simple linear regression was calculated between some pairs of variables.

### Ethical aspects:

Before the study, each participant received detailed information about the investigation’s procedures and objectives. All the subjects signed an informed consent form before participating in the study. The study was approved by the Ethics Committee of the Universidad de San Martin de Porres with Federalwide Assurance (FWA) for the Protection of Human Subjects for International No. 00015320 and the U.S. Department of Health and Human Services (HHS) Registration of an Institutional Review Board (IRB) IRB No. 00003251, approved on 16 December 2022.

## 3. Results

For individuals in both groups, similar characteristics are observed in age, weight, height, and BMI. Male subjects who performed mild physical activity share similar age and height with the homologous group. Female subjects show similar anthropometric characteristics. No statistical differences among anthropometric variables between the two groups were detected (Table 1).

Systolic blood pressure (SBP) was slightly higher in individuals who performed moderate physical activity (Table 2). Female subjects with moderate physical activity presented a higher SBP than the group of the same sex that practiced light physical activity, a pattern similarly observed in male subjects but with less variation (Table 2). Diastolic blood pressure (DBP) remained relatively high in the mild physical activity group (Table 2). DBP was higher in male individuals with mild physical activity compared to the counterpart group with moderate physical activity; the reverse was observed in female subjects (Table 2).

Hemoglobin (Hb) levels were slightly higher in participants with moderate physical activity, and higher in men with light physical activity compared to the other groups. Oxygen saturation (SpO2) was slightly higher in the light physical activity group, with women having higher SpO2 levels than men, regardless of physical activity level. Heart rate was lower in the moderate physical activity group in both sexes (Table 2).

In the left arm, no significant differences were observed in relation to the times of first pain and unbearable pain in the groups that performed light and moderate physical activity; both groups presented similar results, with slight changes in all cases. Resolution time was shorter in the male subjects with physical activity and the female group with mild physical activity compared to the other groups (Tables 3 and 4). No significant differences were observed concerning sex (Tables 5 and 6). Subjects who performed mild physical activity, regardless of sex, perceived the first pain and unbearable pain in a shorter time than subjects who performed moderate physical activity. However, the pain resolution time was shorter in subjects who practiced moderate physical activity (Tables 5 and 6).

**Figure 1.**
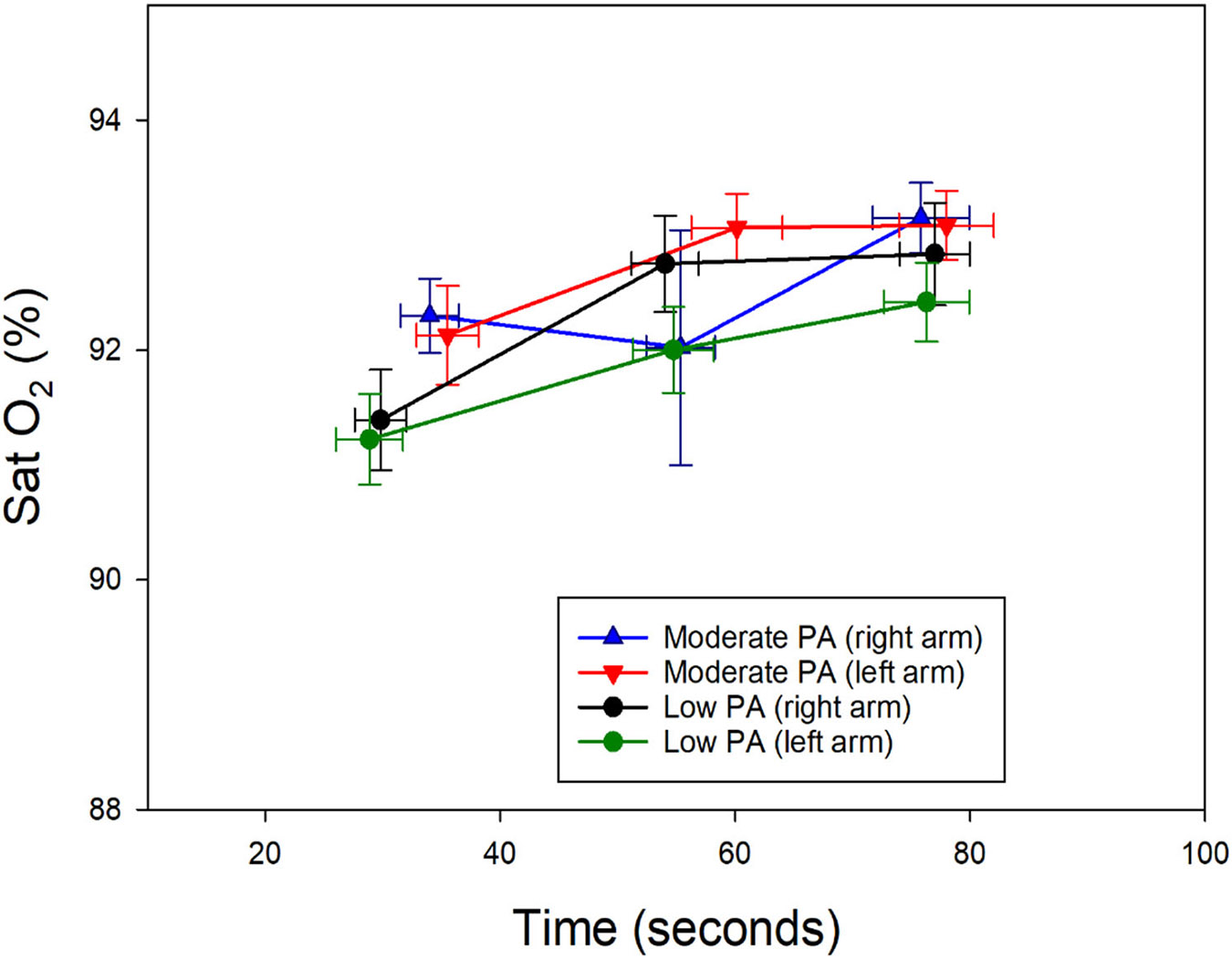
Changes in oxygen saturation during the pain threshold test in the two groups of subjects. Labels for the *X*-axis are time of first pain, time of onset of first pain, excruciating pain, and no pain, over time in seconds. PA: physical activity.

**Figure 2.**
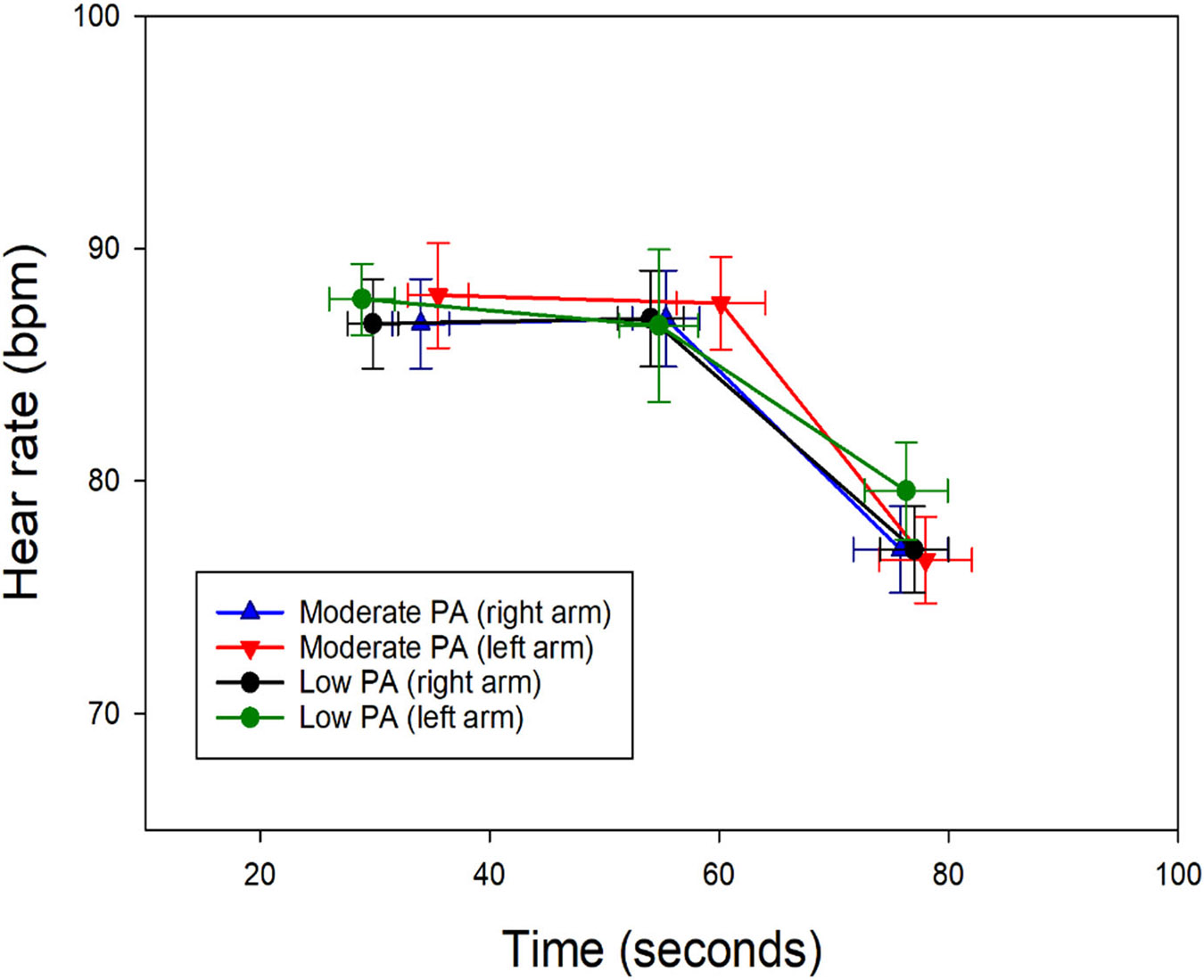
Heart rate changes during the pain threshold test in the two groups of subjects. Labels for the *X*-axis are time of first pain, time of onset of first pain, excruciating pain, and no pain, over time in seconds. PA: physical activity.

In the right arm, the oxygen saturation (SatO_2_) of subjects with moderate physical activity presented with elevated values in the appearance and cessation of pain. Heart rate values were higher in the group of subjects with moderate physical activity at the onset of the first pain, whereas, in subjects with mild physical activity, the values were higher during the onset of unbearable pain and the cessation of pain (Figures 1 and 2).

In the left arm, subjects with moderate physical activity presented with higher SatO_2_ values at the onset and cessation of pain, without major differences during unbearable pain (Figure 1). Heart rate was higher during the onset of first pain in subjects with moderate physical activity; however, the values were higher during the onset of unbearable pain and the cessation of pain in the group of subjects with mild physical activity (Figure 2). SpO_2_ was similar in both groups, with minimal variations between stages of pain perception (Figure 1). Heart rate was slightly lower in subjects performing moderate activity, both during the onset of unbearable pain and in the absence of pain. Heart rate was lower in subjects performing moderate physical activity during unbearable pain and in the absence of pain (Figure 2). Linear regression shows us that SpO_2_ tends to increase with pain intensity, while HR tends to decrease with greater pain intensity; this can be clearly seen in Figures 1 and 2.

## 4. Discussion

One of the most common symptoms perceived by people is pain, so strategies are sought to reduce its perception and alternatives to achieve a better tolerance capacity to this symptom [1]. It is mentioned that physical activity produces changes in the perception of pain [12]. To evaluate whether these conditions allow a better tolerance of the painful sensation, we conducted this study in a hypoxic environment, among people who had lived in this place for a long time, evaluating one group that regularly engaged in physical activity and another whose level of physical activity was lower. This study is the first of its kind, as previous studies have primarily evaluated physical activity, which helps to improve the ability to perceive pain and represents an additional resource for patients living with chronic pain [15]. In addition, in this study, we have evaluated the hypoxic environment, which could also have an additional effect.

In this study, age was slightly higher in people who carried out mild physical activity compared to those who carried out moderate activity; however, this age difference probably had no influence and does not represent a determining factor on the results of the present study due to the approximately one-year difference. Furthermore, all study subjects belonged to the same age group, representing young, apparently healthy individuals without harmful habits.

Subjects who perform moderate physical activity have a slightly higher BMI. This could be explained by a greater muscle mass, likely related to the isometric physical activity performed. This type of isometric exercise is characterized by stimulating the development of muscle mass by activating the mTORC1 pathway, essential for protein synthesis and muscle hypertrophy [15,16]. In addition, it stimulates the proliferation of satellite cells, which fuse with muscle fibers to increase size and functionality [16]. In addition, proteins such as titin act as mechanical load sensors, amplifying the signals that promote muscle growth [17]. Our results suggest that people with moderate physical activity have a lower proportion of adipose tissue compared to those who perform light physical activity. However, a limitation of this study was the absence of an instrument to measure the percentage of body fat in the study subjects, which prevented a precise evaluation of body composition in the study.

The elevated values of SBP and DBP found in male and female subjects (except mild-activity males) who practiced moderate physical activity contradicted findings describing decreased blood pressure in most sports. This finding could be related to the generation of high blood pressure in athletes as a result of exercise-induced hypertension, which is different from the normal physiologic response [18].

Hb levels are slightly higher in participants with moderate physical activity. In addition, Hb levels were elevated in men with mild physical activity compared to the other groups. This finding supports previous evidence [19], although moderate physical activity was not taken into consideration. It was concluded that exercise produces changes in baseline biochemical and hematological values in athletes compared to the general population [19].

SpO_2_ levels were slightly higher in the mild physical activity group. Women also presented with higher SpO_2_ levels compared to men, regardless of the level of physical activity. Studies have shown that, although women tend to have higher oxygen consumption under similar conditions of physical exertion, women maintained higher SpO_2_ levels than men. This difference in the physiological response could be explained by a greater efficiency in oxygen utilization, probably due to factors such as body composition and the greater proportion of fat mass in women, which contributes to better oxygen distribution [20].

The findings obtained in this study are in agreement with previous research, supporting the hypothesis that physical activity, depending on its frequency and intensity, exerts a significant hypoalgesic effect [21,22]. This phenomenon could be explained by the modulation of multiple neurobiological and neuroinflammatory mechanisms. Physical exercise affects pain perception by regulating the release of pro-inflammatory cytokines, attenuating behavioral responses to noxious stimuli, and promoting endogenous anti-inflammatory and analgesic properties [23]. Specifically, in the context of inflammatory pain, exercise has been shown to influence the synaptic plasticity of the anterior cingulate cortex, regulate endocannabinoid systems, modulate spinal excitability in the dorsal horn, and alter the polarization balance of immune cells [23]. These combined mechanisms could explain the differences observed in the onset of pain perception to maximal tolerance, thus reinforcing the role of exercise as an effective tool for pain management.

Consistent with previous research, the results obtained in this study revealed greater sensitivity and tolerance to pain in the dominant upper limb of right-handed subjects [24,25]. This lateralized asymmetry could be attributed to the greater responsiveness of the late-rocapsular division of the right central nucleus compared to its left counterpart. These findings suggest a right hemispheric lateralization in subcortical pain processing, specifically at the level of the thalamus [26].

The differences observed in the perception of pain between men and women are due to complex biological and psychosocial interactions. In the present study, we found a statistically significant relationship between perception and pain, which coincided with previous studies (Tables 3 and 4) [27]. According to the present study, women may have a better capacity for pain tolerance if they practice moderate physical activity. Some studies have shown that biological variables such as hormonal fluctuations and the activation of different receptor systems, including opioid receptors, influence pain sensitivity. In turn, psychosocial factors, such as cultural beliefs and coping patterns, significantly modulate the subjective experience of pain [28]. The results obtained in this study corroborate and support previous multifactorial studies suggesting that pain perception is a highly individualized phenomenon and is influenced by a unique combination of biological and psychosocial factors [29,30].

Although a synergy between exercise and hypoxia on pain sensitivity was previously described [31], the absence of appropriate control groups limits the possibility of comparing our results. Additional studies are required to corroborate these findings and elucidate the mechanisms underlying the pain sensitivity and exercise in a hypoxic condition.

Men tend to have a longer time to the onset of pain and unbearable pain compared to women. Experimental research has shown that men have higher pain thresholds and tolerance compared to women, especially under controlled stimuli (e.g., pressure or thermal) [32]. This difference is attributed to hormonal factors, including higher levels of testosterone, which can modulate endogenous opioid systems and favor greater resistance to pain [33]. Within the group of moderate activity, men have a higher pain threshold and tolerance, which coincides with shorter times of pain exacerbation and resolution. In a study where the relationship between habitual physical activity and experimental pain tolerance was investigated in healthy volunteers, greater habitual physical activity is related to greater experimental pain tolerance, especially in men. Other studies suggested that physical activity can improve the severity of pain [21,34]. In particular, moderate activity was a key factor in increasing the pain threshold, which was explained by the physiological release of endorphins and endocannabinoids during exorcise to reduce the perception of pain, physical activity, and cold pain tolerance [33,35].

In women, moderate physical activity also prolonged the time of unbearable pain but showed faster resolution than mild physical activity. The Tromsø Study has shown that higher levels of physical activity increase pain tolerance in women [2]. This effect is associated with both physiological and psychological adaptations, where moderate exercise could improve the emotional perception of pain and accelerate resolution mechanisms [31,36].

In this study, an increase in SpO_2_ levels was observed globally. In both the study groups, SpO_2_ levels at the appearance of the first pain, unbearable pain, and the state of no pain had no statistical differences (Figure 1). The rising SpO_2_ from increased respiratory frequency can be explained by the interaction of several pathophysiological mechanisms. Specifically, the mechanisms include the activation of chemoreceptors by hypoxia, the production of reactive oxygen species (ROS), and the modulation of the ventilatory response. Arterial chemoreceptors, such as the carotid bodies, play an important role in detecting changes in blood oxygen tension. In a hypoxic situation, these receptors generate mitochondrial signals, such as NADH and ROS, which affect the ion channels of the cell membrane. This causes a depolarization of the cell, allowing the entry of calcium and the release of neurotransmitters. As a result, the respiratory center in the brainstem is stimulated, leading to an increase in respiratory rate [37,38]. The production of ROS also plays an important role in regulating the ventilatory response. During hypoxia, the reduction of NADPH catabolism by the enzyme NOS1 increases the availability of NADPH to NOX, which activates transient receptor potential (TRP) channels through ROS. This activation can increase the sensitivity of central chemoreceptors to CO_2_, resulting in hyperventilation [37,38]. Hypoxia causes a decrease in the partial pressure of arterial oxygen (PaO_2_), which in turn reduces SaO_2_. In response, the respiratory system increases ventilation to try to correct this alteration and improve arterial oxygenation. This mechanism is key to maintaining adequate oxygen transport to the tissues, ultimately affecting oxygen saturation and coincides with the phases of the tests to which the volunteers have been subjected [39,40].

The time of pain perception is consistent between both arms, although the right arm showed a slight advantage in the time of first pain and unbearable pain. This observation is potentially aligned with the plasticity that the nervous system develops with frequent use and exposure to stimuli on a specific side of the body [41].

An increase in heart rate was observed during the appearance of the first pain over baseline values (Figure 2). This is consistent with the effects of the autonomic nervous system to nociceptive stimuli [41]. In the subjects who practiced mild physical activity, the rise occurred progressively until the moment of maximum pain and then decreased, unlike the group of subjects who practiced moderate physical activity, where the highest heart rate was observed during the appearance of the first pain and then decreased. Greater parasympathetic activation associated with intense physical activity may explain this observation relative to a more efficient pain modulation capacity [41,42]. Likewise, increased heart rate in response to pain was also observed in the study subjects who practiced moderate physical activity.

## 5. Limitations

An ideal approach would have been to compare pain tolerance with highly qualified athletes. In this study, it was not possible to include this group because this type of athlete does not exist in the city of Puno. An additional limitation in the present study is that we were unable to use more precise methods that would allow us to measure neuronal conduction velocity, nor any other more sophisticated method that would help to assess pain threshold and perception. The two most common medical devices used to assess pain are the Analgesic Nociception Index, based on heart rate variability, and the Surgical Plethysmographic Index, which is based on plethysmography. Neither of these methods could be used in this study.

## 6. Conclusions

The pain threshold seems to be slightly higher during exposure to a hypoxic environment; however, no statistical significance was observed. In addition, no differences in pain threshold were observed between men and women. Although there was no statistically significant difference, we observed subtle changes in the ischemic pain threshold between altitudes, which points to certain differences associated with exposure to acute hypoxia in young healthy Andean plateau natives when exposed to even higher altitudes.

## Figures and Tables

**Table 1.** Body weight, height, and body mass index (BMI) by sex and level of physical activity.

	Low Physical Activity			Moderate Physical Activity		
	Men (n = 30)	Women (n = 7)	*p*	Men (n = 36)	Women (n = 9)	*p*
Age (y)	23.5 ± 2.5	23.6 ± 1.2	0.845	22.7 ± 1.8	22.2 ± 1.6	0.290
Weight (kg)	67.2 ± 10.4	56.2 ± 5.4	0.066	64.9 ± 10.6	60.2 ± 5.9	0.002
Height (m)	1.7 ± 0.5	1.5 ± 0.5	0.000	1.7 ± 0.5	1.5 ± 0.5	0.000
BMI (kg·m^−2^)	23.9 ± 3.3	23.4 ± 1.9	0.064	23.7 ± 3.4	25.3 ± 2.5	0.583

**Table 2.** Baseline parameters and vital signs in subjects with light physical activity and moderate physical activity by gender.

	Low Physical Activity		Moderate Physical Activity			
	Men (n = 30)	Women (n = 7)	*p*	Men (n = 36)	Women (n = 9)	*p*
Systolic Blood Pressure (mmHg)	107.32 ± 10.3	99.9 ± 12.0	0.081	108.8 ± 10.5	103.9 ± 7.5	0.059
Diastolic Blood Pressure (mmHg)	73.3 ± 7.5	66.9 ± 8.7	0.042	68.5 ± 7.8	69.9 ± 4.2	0.459
Hemoglobin (g/dL)	16.3 ± 1.3	14.9 ± 1.0	0.002	15.9 ± 0.9	15.2 ± 0.9	0.002
Oxygen Saturation (%)	90.7 ± 2.3	91.6 ± 1.9	0.698	90.8 ± 2.1	91.0 ± 2.1	0.698
Heart Rate (bpm)	82.1 ± 11.3	82.1 ± 11.3	0.603	78.3 ± 11.5	79.1 ± 8.4	0.779

**Table 3.** Heart rate, peripheral oxygen saturation, and time course of pain during the test in the right arm.

RIGHT ARM					
		Low Physical Activity	Moderate Physical Activity	Difference	*p*
Time (s)	First pain	30.2 ± 14.1	32.5 ± 15.4	2.3	0.494
	Unbearable pain	54.1 ± 16.4	53.8 ± 19.6	0.3	0.943
	No pain	76.4 ± 18.4	75.2 ± 26.8	1.2	0.825
	Exacerbation time	23.9 ± 8.3	21.3 ± 10.8	2.6	0.250
	Pain resolution time	22.3 ± 10.9	21.4 ± 13.2	0.9	0.744
Sat O_2_ (%)	First pain	91.5 ± 2.6	92.3 ± 2.3	0.8	0.141
	Unbearable pain	92.9 ± 2.3	92.9 ± 2.4	0.0	0.998
	No pain	92.9 ± 2.4	93.2 ± 2.3	0.2	0.685
Heart rate (bpm)	First pain	87.2 ± 10.9	88.8 ± 12.7	1.8	0.513
	Unbearable pain	88.8 ± 12.0	86.9 ± 12.5	1.9	0.487
	No pain	78.2 ± 11.6	74.3 ± 11.8	3.9	0.139

**Table 4.** Heart rate, peripheral oxygen saturation, and time course of pain during the test in the left arm.

LEFT ARM					
		Low Physical Activity	Moderate Physical Activity	Difference	*p*
Time (s)	First pain	27.9 ± 16.8	34.6 ± 18.5	6.7	0.097
	Unbearable pain	53.0 ± 19.6	59.3 ± 24.5	6.3	0.225
	No pain	75.3 ± 22.2	77.1 ± 25.5	1.8	0.749
	Exacerbation time	25.1 ± 12.2	24.7 ± 15.8	0.4	0.894
	Pain resolution time	22.3 ± 11.8	17.8 ± 6.9	4.5	0.023
Sat O_2_ (%)	First pain	91.4 ± 2.4	92.1 ± 2.9	0.8	0.215
	Unbearable pain	92.2 ± 1.9	92.9 ± 2.3	0.7	0.175
	No pain	92.5 ± 1.9	93.1 ± 2.2	0.6	0.222
Heart rate (bpm)	First pain	89.0 ± 8.9	86.7 ± 12.0	2.3	0.352
	Unbearable pain	91.0 ± 13.5	85.6 ± 10.9	5.4	0.041
	No pain	81.8 ± 12.6	75.5 ± 11.5	6.3	0.020

**Table 5.** Heart rate, peripheral oxygen saturation, and time course of pain during the test by gender in the right arm.

RIGHT ARM							
		Low Physical Activity		Moderate Physical Activity			
		Women	Men	*p*	Women	Men	*p*
Time (s)	First pain	31.2 ± 16.5	29.4 ± 12.1	0.728	28.2 ± 8.6	34.7 ± 17.5	0.113
	Unbearable pain	52.4 ± 19.1	55.6 ± 14.0	0.602	46.7 ± 12.5	57.5 ± 21.6	0.039
	No pain	74.6 ± 18.9	78.0 ± 18.4	0.619	70.4 ± 23.3	77.7 ± 28.3	0.315
	Exacerbation time	21.2 ± 7.4	26.3 ± 8.5	0.097	18.5 ± 8.1	22.8 ± 11.7	0.144
	Pain resolution time	22.1 ± 8.1	22.4 ± 13.1	0.955	23.7 ± 15.7	20.2 ± 11.9	0.330
Sat O_2_ (%)	First pain	91.3 ± 2.9	91.6 ± 2.4	0.733	92.4 ± 2.9	92.2 ± 2.1	0.713
	Unbearable pain	93.2 ± 2.3	92.8 ± 2.4	0.590	92.7 ± 3.0	93.1 ± 2.0	0.555
	No pain	92.9 ± 2.4	92.9 ± 2.5	0.992	93.2 ± 2.7	93.1 ± 2.1	0.913
Heart rate (bpm)	First pain	88.8 ± 12.2	85.6 ± 9.9	0.432	91.8 ± 12.7	87.3 ± 12.6	0.196
	Unbearable pain	90.4 ± 14.4	87.4 ± 9.8	0.505	89.5 ± 11.7	85.6 ± 12.8	0.242
	No pain	79.4 ± 10.1	77.2± 13.2	0.608	78.2 ± 14.4	72.4 ± 9.8	0.075

**Table 6.** Heart rate, peripheral oxygen saturation, and time course of pain during the test by gender in the left arm.

LEFT ARM							
		Low Physical Activity		Moderate Physical Activity			
		Women	Men	*p*	Women	Men	*p*
Time (s)	First pain	25.8 ± 18.5	29.8 ± 15.6	0.529	31.7 ± 20.7	36.1 ± 17.3	0.376
	Unbearable pain	50.1 ± 20.8	55.6 ± 18.8	0.449	58.3 ± 26.3	59.8 ± 23.8	0.828
	No pain	74.6 ± 21.3	75.9 ± 23.7	0.883	75.4 ± 26.9	77.9 ± 24.9	0.716
	Exacerbation time	24.3 ± 8.3	25.9 ± 14.9	0.728	26.7 ± 17.9	23.7 ± 14.8	0.487
	Pain resolution time	24.6 ± 12.4	20.3 ± 11.2	0.323	17.1 ± 6.0	18.1 ± 7.4	0.569
Sat O_2_ (%)	First pain	91.1 ± 2.5	91.6 ± 2.4	0.543	92.7 ± 2.8	91.9 ± 2.9	0.297
	Unbearable pain	92.3 ± 2.1	92.1 ± 1.93	0.8	92.9 ± 2.9	93.1 ± 1.9	0.341
	No pain	92.4 ± 1.6	92.6 ± 2.1	0.700	93.2 ± 2.8	93.0 ± 1.9	0.785
Heart rate (bpm)	First pain	89.7 ± 8.9	88.4 ± 9.0	0.701	89.4 ± 9.0	85.4 ± 13.2	0.215
	Unbearable pain	91.6 ± 14.4	90.5 ± 13.1	0.821	86.0 ± 9.7	85.39 ± 11.6	0.837
	No pain	82.8 ± 12.7	80.9 ± 12.9	0.696	76.0 ± 9.9	75.3 ± 12.3	0.821

## Data Availability

The data are available in the database developed by the authors and will be provided to those who request them.
